# Rosiglitazone Affects Nitric Oxide Synthases and Improves Renal Outcome in a Rat Model of Severe Ischemia/Reperfusion Injury

**DOI:** 10.1155/2012/219319

**Published:** 2012-02-15

**Authors:** Boris Betz, Reinhard Schneider, Tobias Kress, Martin Alexander Schick, Christoph Wanner, Christoph Sauvant

**Affiliations:** ^1^Medizinische Klinik und Poliklinik I, Nephrologie, Universitätsklinikum Würzburg, 97080 Würzburg, Germany; ^2^Klinik und Poliklinik für Anästhesiologie, Universitätsklinikum Würzburg, 97080 Würzburg, Germany; ^3^Klinik für Anästhesie und Operative Intensivmedizin, Universitätsklinikum Halle (Saale), 06120 Halle (Saale), Germany

## Abstract

*Background.* Nitric oxide (NO)-signal transduction plays an important role in renal ischemia/reperfusion (I/R) injury. NO produced by endothelial NO-synthase (eNOS) has protective functions whereas NO from inducible NO-synthase (iNOS) induces impairment. Rosiglitazone (RGZ), a peroxisome proliferator-activated receptor (PPAR)-**γ** agonist exerted beneficial effects after renal I/R injury, so we investigated whether this might be causally linked with NOS imbalance. *Methods.* RGZ (5 mg/kg) was administered i.p. to SD-rats (f) subjected to bilateral renal ischemia (60 min). Following 24 h of reperfusion, inulin- and PAH-clearance as well as PAH-net secretion were determined. Morphological alterations were graded by histopathological scoring. Plasma NO_x_-production was measured. eNOS and iNOS expression was analyzed by qPCR. Cleaved caspase 3 (CC3) was determined as an apoptosis indicator and ED1 as a marker of macrophage infiltration in renal tissue. *Results.* RGZ improves renal function after renal I/R injury (PAH-/inulin-clearance, PAH-net secretion) and reduces histomorphological injury. Additionally, RGZ reduces NO*_x_* plasma levels, ED-1 positive cell infiltration and CC3 expression. iNOS-mRNA is reduced whereas eNOS-mRNA is increased by RGZ. *Conclusion.* RGZ has protective properties after severe renal I/R injury. Alterations of the NO pathway regarding eNOS and iNOS could be an explanation of the underlying mechanism of RGZ protection in renal I/R injury.

## 1. Introduction

Acute kidney injury (AKI) is a common clinical complication with uncertain outcome, ranging from complete restitution to high mortality. Ischemia and reperfusion (I/R) injury is a major cause of AKI, frequently occurring as a result of hypotension, hypovolemia, sepsis, or following renal transplantation [1]. I/R injury causes inflammation, renal epithelial cell death, and a reduced organ perfusion and is associated with renal dysfunction [2]. The renal inner cortex and the outer medulla are the predominant morphological sites of I/R injury [2].

Nitric oxide (NO) is fundamentally involved in the pathophysiology of ischemic AKI [1, 3]. NO is thought to exert both protective and deleterious effects depending on the generating enzyme: the generation of NO by inducible NO synthase (iNOS) contributes to renal cell injury due to infiltration with inflammatory cells, by direct DNA damage or by apoptotic effects [1, 4, 5]. On the other hand, a reduced activity of eNOS contributes to renal impairment due to endothelial dysfunction and consecutive renal vasoconstriction [1, 3].

Our group and others demonstrated that nitric oxide pathway is altered in ischemic AKI resulting from (i) a reduced eNOS-expression [6, 7] and (ii) an increased iNOS expression [4, 8] in renal tissue. A correction of this eNOS/iNOS imbalance correlates with an amelioration of renal function after I/R injury [4, 6, 7]. This supports the hypothesis of the eNOS/iNOS balance playing a prominent role in the process of renal I/R injury [9].

Rosiglitazone is the most potent activator of the peroxisome-proliferator-activated receptor gamma (PPAR*γ*). The PPAR*γ* belongs to a nuclear hormone receptor superfamily which regulates transcription by binding to retinoid X receptor that is in turn bound to DNA in various cell types. Activation of PPAR*γ* promotes insulin-stimulated glucose uptake and suppresses proinflammatory responses [10].

Rosiglitazone protects against renal I/R injury in the rat [11]. Additionally, PPAR*γ* agonists reduce inflammatory response during renal I/R injury [11, 12]. However, the underlying mechanisms are not fully investigated yet. Using inflammatory animal models, rosiglitazone significantly reduces the expression of iNOS and the generation of NO [13–15]. In addition, there is evidence that rosiglitazone may additionally affect the protective branch of NO production mediated by eNOS in I/R injury [16]. Consequently, we hypothesized that rosiglitazone may exert its protective action in renal I/R damage via influencing both iNOS and eNOS.

We tested this hypothesis in our well-established rat model of I/R injury [4, 5] with rosiglitazone applied during the ischemic period.

Effects on renal functional outcome were monitored by glomerular filtration rate (GFR) and renal plasma flow (RPF). Histomorphological damage was examined and scored for its severity. The influence of rosiglitazone on the imbalance of eNOS and iNOS expression and NO generation were analyzed. Cellular inflammatory responses visualized by cortical ED-1-positive cell infiltration as well as effects on apoptotic cleaved caspase 3 (CC3) protein expression were determined.

## 2. Materials and Methods

### 2.1. In Vivo Experimental Procedure

All animal care and experimental procedures performed in this study were in accordance with the German laws for animal protection.

Sprague-Dawley (CD) rats (♀, 200–250 g body weight) were obtained from Charles River Wiga GmbH (Kissleg, Germany). Anesthesia was performed by intraperitoneal application of xylacin hydrochloride (10 mg/kg body weight) and ketamine (100 mg/kg body weight). All operative procedures were performed on thermoregulated heating boards to maintain body temperature at 37.0°C. Postoperative pain relief was assured by subcutaneously applied tramadol (0.05 mg/kg body weight); postoperative dehydration was prevented by subcutaneous administration of an additional 1.0 mL of 0.9% NaCl. Animals were divided into the five following subgroups:

- *control group: reflecting day 0 (control);*
- *sham group: supplementation with saline (sham);*
- *sham group: supplementation with rosiglitazone (sham + RGZ);*
- *clamping group: supplementation with saline (clamp);*
- *clamping group: supplementation with rosiglitazone (clamp + RGZ).*

I/R injury was induced in rats by bilateral clamping of the renal arteries for 45 and 60 minutes as described previously [4, 5]. The same procedure was performed in sham animals without the bilateral clamping process. Rosiglitazone (5 mg/kg ip) or saline, respectively, were given intraperitoneally before the end of the clamping period (or after sham operation) to assure immediate delivery into the kidney right at the beginning of reperfusion and to exclude preconditioning pretreatment effects of rosiglitazone prior to ischemia. Control animals remained completely untreated.

### 2.2. Measurement of Clearances of Inulin (GFR) and PAH (RPF)

Inulin and PAH clearances were calculated by using inulin (fluorescein-isothiocyanate inulin) or paraaminohipurate (both from Sigma Aldrich, St. Louis, MO, USA) concentrations in plasma and urine samples as recently described in detail [5].

### 2.3. Organ Preparation and Tissue Harvesting

After perfusion under pressure-controlled conditions (100 mmHg) with ice-cold Krebs buffer [in mM: 118 NaCl, 25 NaHCO_3_, 4.8 KCl, 1.2 KH_2_PO_4_, 1.2 MgSO_4_, 11 glucose, and 1.5 CaCl_2_(2H_2_O)] for 20 s, samples of renal cortex including parts of the outer medulla were removed from whole kidney, snap-frozen in liquid nitrogen, and stored at −80°C.

### 2.4. Protein Immunoblotting

For western blot analysis, frozen kidney cortices were mechanically homogenized and dissolved in lysis buffer containing 150 mM NaCl, 10 mmol/L Tris (Base) pH 7.3, 1% Nonidet P40 (Igepal), 0.1% SDS, 1% sodium-desoxychloric acid, 0.1% Triton X-100, 1 mM EDTA, 184 mg/L sodium orthovanadate, and 0.1% protease inhibitor cocktail (100 mM AEBSF-HCl, 80 mM aprotinin (bovine lung, crystalline), 5 mMbestatin, 1.5 mM protease inhibitor E-64, 2 mM leupeptin hemisulfate, and 1 mM papstatin A). Total protein was measured in each sample using the Bradford method.

Samples of protein (30 *μ*g) were analyzed by Western blot with the cleaved caspase 3 polyclonal antibody (diluted 1 : 250, Cell Signaling, Boston, MA, USA). Blots were incubated with horseradish peroxidase-conjugated anti-rabbit IgG (1 : 2,000; Dako, Hamburg, Germany) and developed using a chemiluminescence kit (ECL Plus, Amersham, GE Healthcare, Buckinghamshire, UK) following the manufacturer's instruction. Each gel was specifically corrected for loading control using *β*-actin (diluted 1 : 50.000) as reference. All western blots were analyzed densitometrically by using the ImageJ software (http://rsbweb.nih.gov/ij/) and plotted with SigmaStat/SigmaPlot software (Systat Software, San Jose, CA, USA). 

### 2.5. Real-Time RT-PCR

RNA from kidney cortices were extracted using the Qiagen RNA Isolation Kit (Qiagen, Hilden, Germany). RNA concentration was determined, and cDNA was synthesized using the iScript cDNA synthesis kit (Bio-Rad Laboratories Inc, Benicia, CA, USA) according to the manufacturer's instructions. In brief, RT-PCR was performed according to the iQ SYBR-Green Supermix RT-PCR system protocol (Bio-Rad Laboratories Inc, Benicia, CA, USA). Initial denaturation was performed at 95°C for 3 min. PCR amplification was performed as described in respective references. iNOS and eNOS were determined as described by Cañuelo et al. [17]. For iNOS, the primers were 5′-GCA GGT TGA GGA TTA CTT CTT CCA-3′ (sense) and 5′-GCC CTT TTT TGC TCC ATA GGA AA-3′ (antisense), resulting in an 94-bp RT-PCR product. For eNOS, the primers were 5′-CAC ACT GCT AGA GGT GCT GGA A-3′ (sense) and 5′-TGC TGA GCT GAC AGA GTA GTA C-3′ (antisense), resulting in a 109-bp RT-PCR product. For *β*-actin, the primers were 5′-TCT ACA ATG AGC TGC GTG TG-3′ (sense) and 5′-TAC ATG GCT GGG GTG TTG AA-3′ (antisense), resulting in an 129-bp RT-PCR product. The RT-PCR products generated with primers for iNOS and eNOS were tested by sequencing (MWG Biotech, München, Germany) and were found to represent the predicted parts of the respective mRNAs. The RT-PCR products were tested for correct size by agarose gel electrophoresis and melting point analysis. Quantification was performed using the ΔΔC_T_ method with *β*-actin as a reference gene. Expression in control animals was normalized to 100.

### 2.6. Detection of Nitric Oxide

Nitrate and nitrite (NO*_x_*) in the plasma level was determined to measure the NO generation. Detection of NO*_x_* was performed using the nitrate/nitrite colorimetric assay kit obtained from Cayman Chemical Company (Ann Arbor, MI, USA) in a 96-well plate according to the manufacturer's protocol.

### 2.7. Detection of Invading Monocytes/Macrophages

Immunofluorescence detection of invading ED-1-positive cells was done as previously described in detail [18]. In brief, cryostat sections (5 *μ*m) were fixed in PBS buffer with 4% paraformaldehyde at a temperature of 4°C for 10 min. After being rinsed with PBS, buffer sections were blocked with 50 mM NH_4_Cl for another 10 min, followed by another rinsing in PBS. Additionally, sections were incubated with 0.1% Triton X-100 in PBS buffer for 10 min. Finally, they were blocked with 10% donkey serum in 0.1% Triton X-100 in PBS buffer for 1 h. Subsequently, the anti-rat macrophage ED-1 (CD68) antibody (diluted 1 : 400; Acris BM 4000, Herford, Germany) was incubated in 10% donkey serum in PBS buffer, followed by donkey anti-mouse Cy3-conjugated secondary antibody (diluted 1 : 500; model 715-165-151; Dianova, Hamburg, Germany) in 10% donkey serum for 1 h. After a last rinse in PBS and H_2_O, ED-1 visualisation in renal tissue sections was performed using an epifluorescence microscope (NIKON Eclipse TE 2000-S, Tokyo, Japan). Finally, the quantity of ED-1 positive cells was determined by calculation of the mean value from the manually counted number in three randomly defined visual fields of each renal section.

### 2.8. Histopathology and Scoring for the Severity of Injury

Cryostat sections (5 *μ*m) were fixed in acetone (Baker, Holland). Sections were stained with periodic acid-Schiff (Roth, Germany) (PAS) and counterstained with Hematoxylin Solution, Gill No. 3 (GHS 332-1l). Histopathologic alterations of the kidney (interstitial edema, ablation of tubular epithelium from the basement membrane, ablation of the brush border from the epithelium of the proximal tubuli, and cell death) were analyzed semiquantitatively by a blinded investigator according to a score (0–3) which was described previously in detail [19]. Mean values from the separate scores were taken together as total injury score.

### 2.9. Data Analysis and Materials

Data are presented as means ± SEM. The *n* values are given in the text or in the figures. *n *equals the number of rats or experiments (RT-PCR, western blot) with tissue or tissue extractions from distinctive rats.

 Statistical significance was determined by unpaired Student's *t*-test for all experiments except for the measurement of NO*_x_* and the histopathologic scores. For NO*_x_*-levels statistical significance was determined by ANOVA test followed by pairwise multiple comparison using the Student-Newman-Keuls-Test. For histopathologic scores differences were assessed using Kruskal-Wallis-Test performed with SPSS 19.0 statistical software (Systat Software, San Jose, CA, USA). Differences were considered statistically significant when *P* < 0.05.

Rosiglitazone (RGZ, AVANDIA) was purchased from GlaxoSmithKline (Brentford, Middlesex, UK). Tramadol (Tramal ) was from Grünenthal GmbH (Aachen, Germany), xylacin hydrochloride (Rompun ) was from Bayer AG (Leverkusen, Germany), and ketamine (Ketanest ) was from Pharmacia & Upjohn Inc (Bridgewater, NJ, USA). If not indicated otherwise, all substances were diluted in 0.9% NaCl (w/v). If not stated otherwise, chemicals were from Sigma Aldrich (St. Louis, MO, USA).

## 3. Results

### 3.1. Rosiglitazone Improves Parameters of Renal Function in Severe Renal I/R Injury

**Figure 1:**
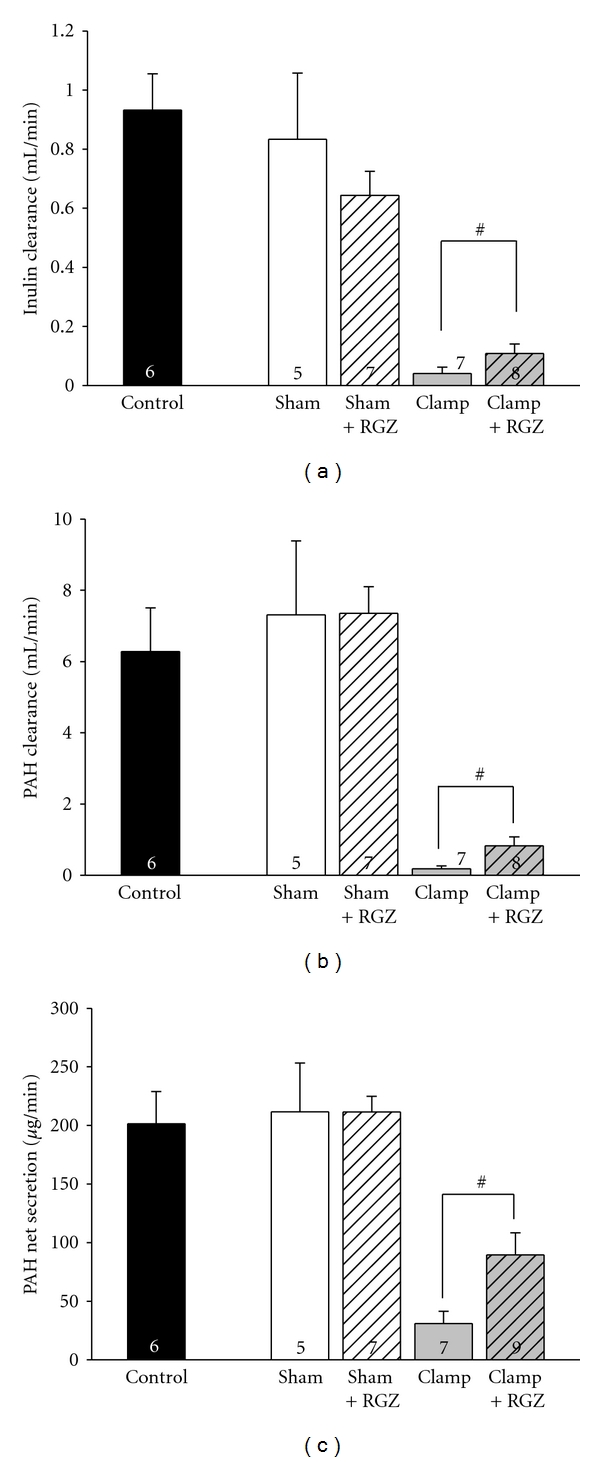
Effect of 5 mg/kg rosiglitazone (RGZ) on parameters of renal function in Sprague-Dawley rats in renal I/R injury. After either a sham operation or a bilateral clamping of the renal arteries for 60 min, rosiglitazone was applied i.p. or not, and parameters of renal function were determined 24 h afterwards. Parameters were additionally determined in untreated control rats. *n *as indicated. ^#^Statistically significant difference as indicated. (a) Renal clearance of inulin was determined as a measure of GFR as described in Section 2 (GFR = (*I*_*U*_ × *V*_*U*_)/(*I*_*P*_ × *t*), where *I_U_* is inulin concentration in urine, *I_P_* is inulin concentration in plasma, *V_U_* is urine volume, *t* is time of measurement, and GFR is glomerular filtration rate). (b) Renal clearance of PAH was determined as described in Section 2 (PAH clearance = (PAH_*U*_ × *V*_*U*_)/(PAH_*P*_ × *t*), where PAH*_U_* is PAH concentration in urine, PAH*_P_* is PAH concentration in plasma). (c) Renal net secretion of PAH (PNS) was determined as described in Section 2  {PNS = [(PAH_*U*_ × *V*_*U*_)/*t*]−[GFR × PAH_*P*_]}.

Comparison of control animals (reflecting day 0) with sham-treated animals revealed no difference of both GFR and RPF (measured by inulin and PAH clearance) (Figures 1(a), and 1(b)). This indicates that the surgical procedure itself had no effect on renal function. Moreover these results show the reliability of the applied method by being in good accordance with the results published by our group before [4, 5]. In sham-operated rats, rosiglitazone application neither affected inulin clearance nor PAH clearance 24 h after intervention. As expected, severe I/R injury with a 60 min ischemic time caused a significant decline in inulin clearance reflecting a marked glomerular dysfunction (Figure 1(a)). Rosiglitazone attenuated the decrease of inulin clearance. Nevertheless, inulin clearance was still substantially lower compared to sham-treated animals or control.

 A similar profile was observed for PAH clearance. Rosiglitazone application provided a threefold improvement of PAH clearance in severe I/R injury when compared to untreated postischemic animals (Figure 1(b)). However, PAH clearance was still substantially diminished despite rosiglitazone treatment in severe I/R injury in comparison to sham-treated animals or controls. 

In summary, the distinct decrease of inulin and PAH clearance in severe renal I/R damage was moderately but significantly attenuated by rosiglitazone.

### 3.2. Rosiglitazone Fails to Improve Renal Function Significantly in Moderate I/R Injury

In an in vivo model of renal I/R injury caused by an ischemic period of 45 min, treatment with rosiglitazone showed a trend towards improvement of renal functional parameters (GFR, RPF) but failed to reach statistical significance (data not shown). Possibly the expected protective effects were not strong enough to induce a significant functional amelioration as discussed later. Increasing the dose of rosiglitazone was in our opinion no favorable option with respect to the overall daily dose recommended for humans (8 mg/d).

Therefore, we further analyzed the data obtained from our model of severe ischemic AKI with extended ischemic period of 60 min.

### 3.3. Rosiglitazone Improves PAH Net Secretion (PNS) in Renal I/R Injury

Since PAH clearance is dependent on both renal perfusion and net secretion of PAH via proximal tubular cells, PAH-net secretion (PAH-NS) was subsequently investigated to clarify the mechanism for the increase of PAH clearance. Likewise, reduction of PAH-NS was attenuated in part by rosiglitazone in renal I/R damage (Figure 1(c)). This indicates an improvement proximal tubular cell function mediated by rosiglitazone. In sham-treated animals neither rosiglitazone nor vehicle significantly influenced PAH-NS.

Rosiglitazone induced a more intense increase of PAH clearance (+330%) compared to PAH-NS (+210%) following severe I/R injury. This indicates that the rise of PAH clearance is not exclusively based on an increase of PAH-NS (and therefore on proximal tubular cell function) but is also due to improvement of renal perfusion as well.

### 3.4. Rosiglitazone Reduces Histomorphological Damage in Severe Renal I/R Injury

**Figure 2:**
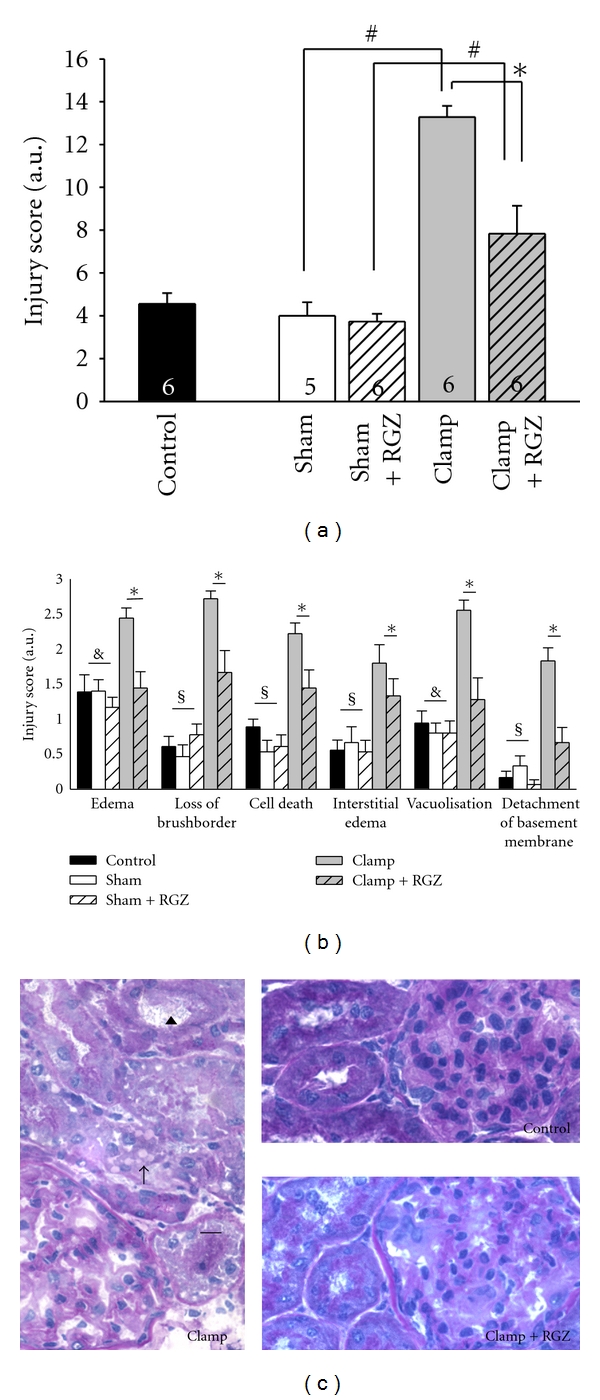
Effect of rosiglitazone (RGZ) on morphological alterations in the kidney of Sprague-Dawley rats in renal I/R injury. Renal cryostat sections were stained with periodic acid-Schiff (PAS). Injury was graded by a score as described in Section 2. (a) Total injury score (mean values from Figure 2(b) were taken together). *n *as indicated. ^#^, *Statistically significant difference as indicated. (b) Single criteria of histopathologic investigations are shown. *n* from 4 to 6 for each group. *Statistically significant difference (*P* < 0.05) between clamp versus clamp + RGZ; ^$^statistically significant difference (*P* < 0.05) between control, sham, sham + RGZ versus clamp and clamp+RGZ; statistically significant difference (*P* < 0.05) between control, sham, sham + RGZ versus clamp alone. (c) Representative sections of images of the renal cortex after PAS staining are shown. Control group, clamp group, and clamp + RGZ group as indicated on the sections. Some of the lesion criteria are exemplified. Arrowhead points to loss of the brush border. Arrow shows vacuolization in epithelial cells of the tubuli. Lines without arrowheads show tubular epithelial cells with defragmented nuclei.

Histopathologic observations revealed that total injury score was significantly increased in both clamping groups compared to the other groups. Treatment with rosiglitazone in the severe I/R injury model resulted in a significant reduction of histomorphological damage. Formation of cell edema and vacuolization were significantly increased only in the clamping group without rosiglitazone. Increased interstitial edema, loss of brush border in the proximal tubular cells, cell death, and detachment of basement membrane were visible in both clamping groups, but significantly reduced in rosiglitazone treated clamping group compared to untreated clamping group (Figures 2(a)–2(c)).

### 3.5. eNOS mRNA Is Upregulated by Rosiglitazone in Renal I/R Injury

**Figure 3:**
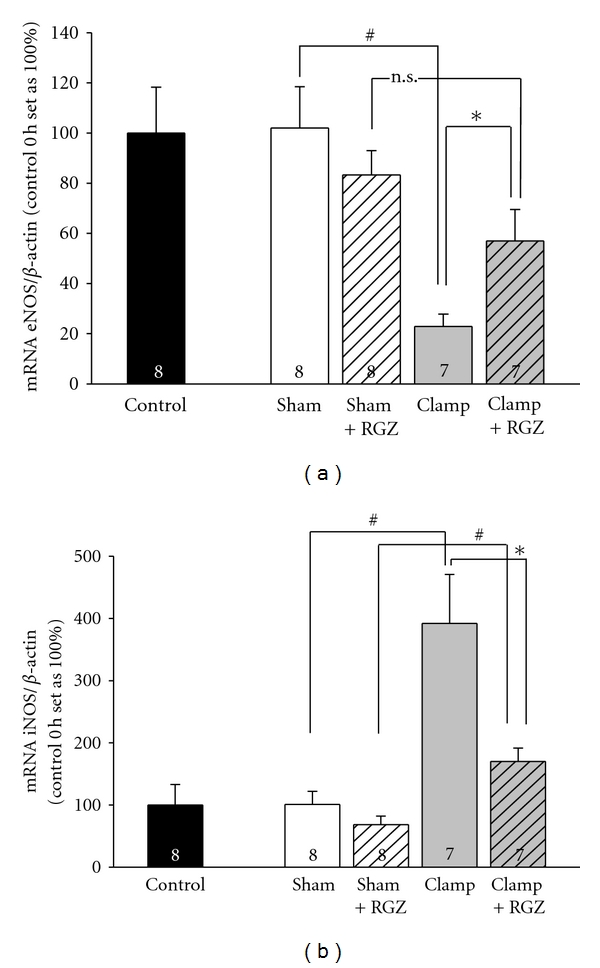
Effect of 5 mg/kg rosiglitazone (RGZ) on the mRNA levels of (a) endothelial and (b) inducible NO-Synthase (eNOS and iNOS) in Sprague-Dawley rats in renal I/R injury. Total RNA was generated from kidney cortex. Real-time PCR against eNOS, iNOS, and *β*-actin was performed as described in Section 2. Quantification was performed using the DDC_ T_ method using *β*-actin as a reference gene, and expression in untreated control animal (control 0 h) was normalized to 100. *n *is given in the respective bars. (a) Effect RGZ on the mRNA levels of eNOS. The amount of eNOS mRNA signal normalized to the respective signal from *β*-actin. ^#^,* Statistically significant difference as indicated. n.s.: Statistically not significant. (b) Effect of RGZ on the mRNA levels of iNOS. The amount of iNOS mRNA signal normalized to the respective signal from *β*-actin. ^#^,*Statistically significant.

It is well known that eNOS plays an important role in the regulation of renal perfusion [1, 3]. Additionally, it is known that PPAR*γ* agonists can induce renal eNOS expression in rats [20]. In vehicle-treated animals, I/R damage led to a significant reduction of eNOS mRNA expression in renal cortex when compared to controls or sham-treated animals 24 h after intervention. Compared to vehicle, eNOS RNA expression was less diminished with rosiglitazone application in severe I/R injury. There was no significant difference between rosiglitazone-treated clamp animals and sham animals or controls. (Figure 3(a)).

### 3.6. iNOS mRNA Is Downregulated by Rosiglitazone in Renal I/R Injury

iNOS is known to play an important role in the pathophysiology of renal I/R injury [3]. Chen et al. showed that iNOS-mRNA is substantially upregulated in renal I/R injury [8]. In our study, iNOS mRNA cortical expression considerably increased (400%) in renal I/R injury period when compared to sham-treated animals or control. Rosiglitazone almost totally abrogated this distinct iNOS increase (Figure 3(b)), resulting in iNOS mRNA expression similar to sham-treated animals or control. Rosiglitazone had no significant effect on iNOS mRNA expression of sham-treated animals.

### 3.7. Rosiglitazone Diminishes NO_x_ Production in Renal I/R Injury

**Figure 4:**
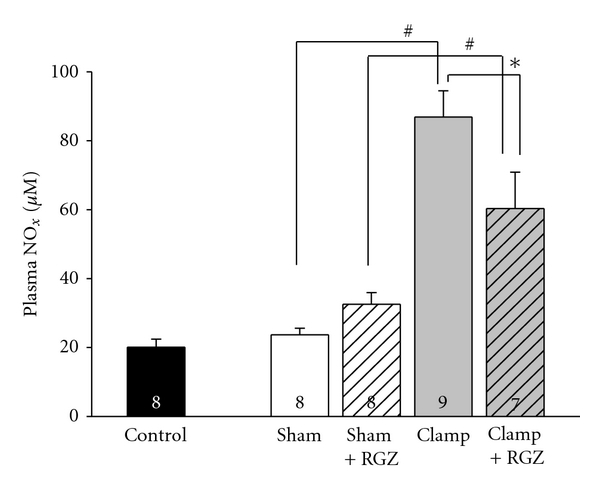
Effect of 5 mg/kg rosiglitazone (RGZ) on nitrite/nitrate (NO*_x_*) concentration in plasma of Sprague-Dawley rats in renal I/R injury. NO*_x_* as a measure of NO was detected by Griess-reaction as described in Section 2. NO*_x_* concentration is given in *μ*M. *n *is given in the respective bars. *, ^#^ Indicate statistically significant difference with *P* < 0.05.

Schwartz et al. demonstrated that iNOS mediates regulation of eNOS via the NOS product NO [21]. Therefore, concentration of both nitrite and nitrate as an established marker of NO generation was determined. Severe renal I/R injury resulted in a significant increase of plasma NO*_x_* when compared to sham-operated animals (Figure 4). Rosiglitazone decreased the elevated NO*_x_* levels in severe I/R injury. There was no increase of NO*_x_* in sham-treated animals. Noteworthy, the NO*_x_*-levels correlate with the iNOS mRNA expression, indicating that iNOS is the major source of NO*_x_* in severe I/R injury.

### 3.8. Cleaved Caspase 3 Expression Is Downregulated by Rosiglitazone in Severe Renal I/R Injury

**Figure 5:**
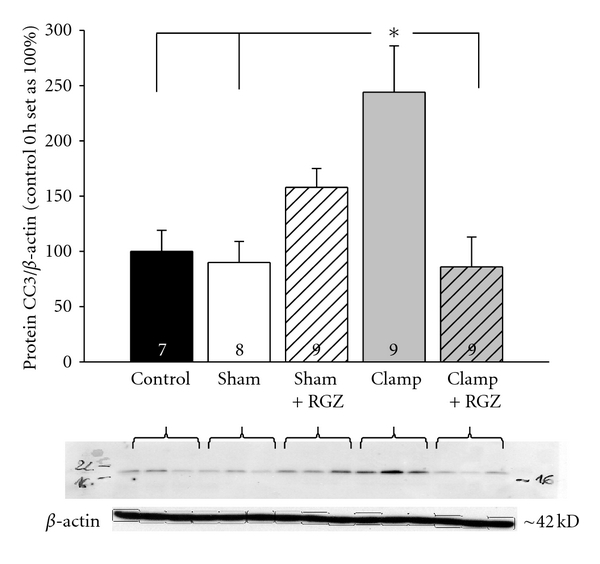
Effect of 5 mg/kg rosiglitazone (RGZ) on the relative expression of cleaved caspase 3 (CC3) in renal cortex of Sprague-Dawley rats in renal I/R injury. Antibody against CC3 recognized a band in the range of 18 kDa; the anti-*β*-actin antibody recognized a band at 42 kDa. The amount of CC3 Western blot against CC3 and *β*-actin was performed as described in Section 2. Western blot signal was normalized to the respective signal from *β*-actin. *Statistically significant difference with *P* < 0.05; *n *as indicated.

The protein expression of CC3 is a marker of cell apoptosis [18]. The expression of cleaved caspase 3 (CC3) is increased in severe I/R injury when compared to sham-treated animals or control (Figure 5). This is in good accordance with results from Raff et al. [18]. This increase of CC3 in severe I/R injury was totally abrogated by the application of rosiglitazone (Figure 5). Notably, rosiglitazone led to an opposite CC3 regulation with a significant increase of CC3 expression in sham-treated animals.

### 3.9. Invasion of ED-1 Positive Cells Is Reduced by Rosiglitazone in Severe Renal I/R Injury

**Figure 6:**
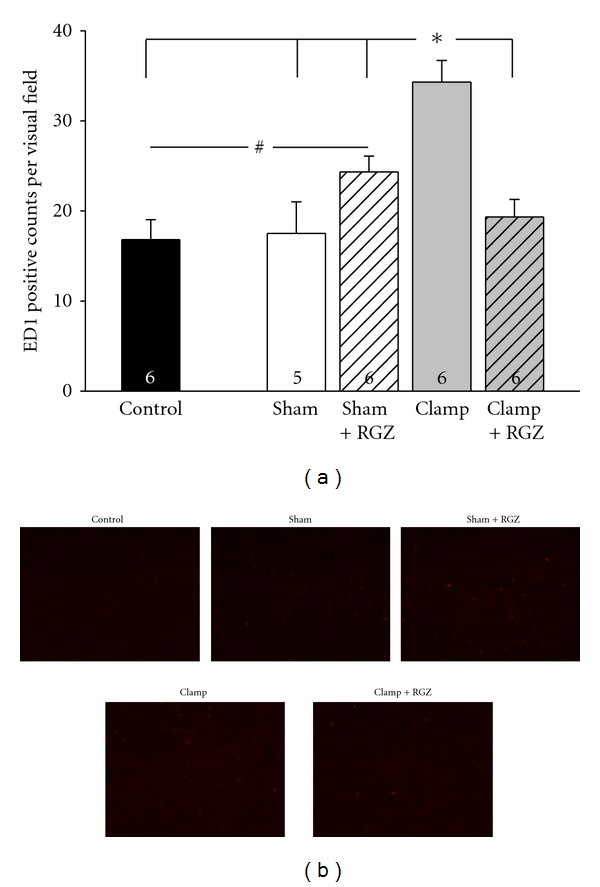
Effect of 5 mg/kg rosiglitazone (RGZ) on invasion of ED-1-positive cells (monocytes/macrophages) in renal cortex of Sprague-Dawley rats in renal I/R injury. (a) ED-1-positive cells were detected in renal cryostat sections by immunofluorescence as described in Section 2. (a) Macrophage/monocyte invasion is given as the amount of ED-1-positive cells per visual field. *n* as indicated. * and ^#^ indicate statistically significant difference with *P* < 0.05. (b) Representative immunohistochemistry images of the renal cortex (magnification 1 : 10) after ED1 staining are depicted. Respective groups as indicated on the sections.

It is well known that monocytes/macrophages invade the renal cortical tissue in I/R injury as a part of a postischemic inflammatory response. The detection of ED-1-positive cells reflecting invading monocytes/macrophages in renal I/R injury is well described [18]. The amount of renal cortical ED-1-positive cells was determined as an estimate of renal inflammatory response in severe I/R injury. Here, I/R injury significantly increased the amount of ED-1-positive cells, whereas rosiglitazone averted the cell infiltration indicating a reduction of renal inflammatory response (Figure 6). In sham-treated animals both CC3 expression (as already mentioned above) and ED-1 infiltration were slightly but significantly increased when rosiglitazone was applied. At the moment we can only speculate about proapoptotic and inflammatory responses due to rosiglitazone. However, since there is no difference in renal function among the sham-treated group versus controls 24 h after intervention, these proapoptotic and inflammatory effects of rosiglitazone seem not to be functionally relevant.

## 4. Discussion

In the present study, the effect of the PPAR*γ* agonist rosiglitazone was investigated in a well-established model of ischemic acute kidney injury [4–6, 18]. The application of rosiglitazone tended to result in an improvement of renal functional parameters regarding an ischemia period of 45 min. However—in contrast to other studies [11, 12]—this trend was not significant. In the related studies [11, 12] the protocol included repeated applications of rosiglitazone and a shorter period of reperfusion (6 h) in comparison to this study (24 h). Moreover, both studies [11, 12] used male rats. In renal I/R injury there is a sexual dimorphism with female rats being much more resistant to ischemia most probably due to their hormonal status [22].

Consequently we speculate that the protective effect of rosiglitazone may depend on the extent of renal damage. In our study, the use of more resistant female rats may have blurred the effect of rosiglitazone in renal I/R injury with an ischemic period of 45 min. Increasing the dose might have rendered the protective effect of rosiglitazone significant in our model of renal I/R injury with an ischemia period of 45 min. However, as already mentioned, the body weight adapted dose was already in the upper range of recommended dose in human. This is especially important as impaired renal function implies the risk of drug accumulation as renal tubular excretion of rosiglitazone is mediated by organic cation transporter 1 which depends on renal function [23]. Thus, we did not increase the dose of rosiglitazone but enlarged renal damage by extending the ischemic period.

As far as we know, we are the first to investigate the effect of rosiglitazone in a model of severe kidney injury defined by an ischemia period of 60 min and a reperfusion period of 24 h. The administration of rosiglitazone in severe renal I/R injury almost tripled GFR (as determined by inulin-clearance). However, in comparison to sham-treated animals, renal function was still markedly reduced despite rosiglitazone treatment.

Besides GFR, rosiglitazone improved PAH clearance (reflecting RPF) in severe renal I/R injury. Since PAH net secretion itself is ameliorated by rosiglitazone, it is hardly possible to determine to what extent rosiglitazone improves renal perfusion from our data. However, in postischemic animals treated with rosiglitazone the PAH clearance increased to a higher extend (~350% compared to vehicle) as compared to PAH net secretion (~200% compared to vehicle). This is indicative for improved vascular function due to rosiglitazone. For the first time, it is demonstrated that a PPAR*γ* agonist improved PAH net secretion. PAH is secreted by the organic anion transport system located in the renal proximal tubular cells. Since the expression of the rate-limiting transporters for PAH secretion (OAT 1 and 3) is not changed by rosiglitazone (data not shown) it seems obvious that the amelioration of PAH net secretion is due to reduced tubular damage and improved tubular integrity.

Corresponding to the amelioration of functional markers we observed reduced histomorphological damage in severe I/R injury when rosiglitazone was applied. This is in good accordance with previous studies [11, 12].

In renal I/R injury cellular damage is mediated by inflammation and cell apoptosis [1, 2]. Indeed, analyzing inner renal cortex and outer medulla, the expression of cleaved caspase 3 which is an established marker of apoptosis [18] was elevated. Moreover the number of ED-1-positive inflammatory cells was increased in our model of severe I/R injury. The application of rosiglitazone inhibited the rate of apoptosis and attenuated renal inflammation. Likewise, previous studies demonstrated similar results investigating the effect of rosiglitazone on renal ischemia [11] or on cisplatin-induced nephrotoxicity in mice [24].

**Figure 7:**
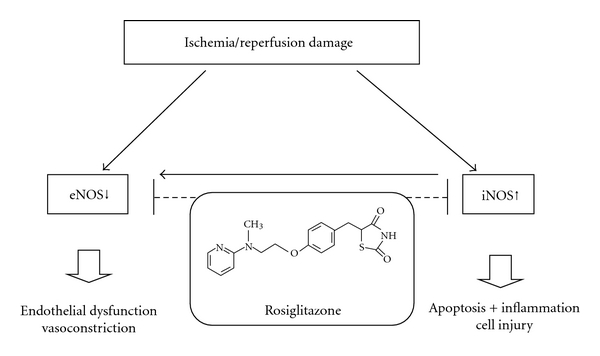
Schematic figure of factors contributing to renal dysfunction in ischemia/reperfusion injury. The downregulation of eNOS causes endothelial dysfunction and vasoconstriction. The upregulation of iNOS in acute kidney injury is associated with an increase in cell apoptosis and inflammatory cell damage. Moreover, elevated levels of iNOS directly suppress the expression of eNOS. The application of rosiglitazone in ischemic acute kidney injury and reperfusion may help to correct the imbalance of eNOS/iNOS expression.

In acute kidney injury iNOS is fundamentally involved in the process of kidney damage by inflammation and apoptosis (Figure 7). The inhibition of iNOS activity (or the absence of iNOS itself in KO-mice) ameliorates renal I/R damage in vivo [25, 26]. In the present study, we report that severe I/R injury causes a strong increase of iNOS mRNA expression subsequently followed by the increased production of NO. This is in good accordance with previous studies [4, 8, 12, 27]. We show that in severe renal I/R injury the application of rosiglitazone not only inhibited generation of apoptosis and attenuated inflammation but also abolished the induction of iNOS and the production of NO. This is in good accordance with in vitro studies reporting that attenuation of iNOS expression is associated with a reduction of parameters of inflammation and apoptosis as well as reduced proximal tubular cell damage [28, 29]. Chatterjee et al. report that the PPAR*γ* agonist 15-deoxy-Δ^12,14^-prostaglandin J_2_ reduces iNOS induction and NO production in rat proximal tubular cells [12]. iNOS is expressed in renal tubular cells and in invading inflammatory cells like macrophages [1, 2, 14, 29]. From our data, it is not possible to discriminate the origin of iNOS expression. However, Schrier et al. and many studies [1, 4, 8, 13, 15] indicate that the deleterious effect of increased iNOS expression in tissue is mainly independent from its origin. In the present study an attenuated increase of iNOS expression can be demonstrated even when infiltration of inflammatory cells is almost completely inhibited by rosiglitazone. Assumedly, the induction of iNOS is mediated by both the infiltration of inflammatory cells and an upregulation in tubular cells itself. In summary, rosiglitazone seems to reduce tubular dysfunction and improve renal outcome by affecting iNOS expression and its inflammatory and apoptotic responses in this model of severe I/R injury.

The regulation of eNOS and its product NO plays a major role in renal perfusion and glomerular function in physiological and pathophysiological conditions [3, 9]. In the present study, the expression of eNOS is reduced in I/R injury [6, 7]. The overexpression of iNOS and the inflammatory response may result in a reduced expression of eNOS [21, 30]. Consequently, the iNOS upregulation most likely contributes to eNOS reduction in the present model as well. The administration of rosiglitazone attenuated the downregulation of eNOS mRNA expression which was accompanied by an amelioration of GFR and RPF. A correlation of eNOS expression to renal function following kidney damage was reported by previous studies [6, 7]. Concerning the effect of PPAR*γ* agonists on eNOS expression in the literature, Song et al. demonstrate an increase of renal eNOS expression by treatment with rosiglitazone [20]. However, other studies suggest that there is no influence of PPAR*γ* agonists on eNOS expression [31, 32]. The conflicting data may result either from the use of PPAR*γ* agonists less potent than rosiglitazone or from the analysis of different target organs (heart, vascular system). In summary, we are the first to investigate the positive influence of a PPAR*γ* agonist on eNOS expression which is most probably associated with an improvement of renal vascular function in renal I/R injury.

We show that the renal expression levels of eNOS and iNOS are contrariwise altered in severe renal I/R injury. This is in good accordance with the literature, significantly attenuated eNOS activity (87% decrease) and augmented iNOS activity (80% increase) in renal cortex of rats subjected to I/R injury was described previously [33]. As already mentioned in Section 1, iNOS and eNOS have different effects in renal I/R injury. Increased expression of iNOS plays a key role in cytotoxicity by inflammation response, necrosis, and apoptosis (Figure 7). Moreover, increased iNOS expression is described to reduce eNOS expression [21, 30]. Reduced eNOS expression impairs vascular function and renal perfusion. In the present study, it has been demonstrated for the first time that rosiglitazone exerts its beneficial effects on renal function by both lowering increased iNOS expression and raising reduced eNOS expression (Figure 7).

Both eNOS and iNOS produce NO. In the present study, the serum level of nitrite and nitrate as markers of NO level are increased after ischemic acute kidney injury (iAKI) while the application of rosiglitazone significantly reduced—but not normalized—the levels. From our data, it is not possible to discriminate between eNOS-derived and iNOS-derived NO production. However, NO production of active inducible NOS is known to far exceed the NO production of eNOS [34]. In addition, NO production by eNOS is reduced in iAKI [1, 3, 6, 9]. Therefore, the rise of NO plasma level is most likely attributed to iNOS generation in I/R injury. This assumption is in good accordance with data from other studies [12, 27]. Moreover, a reduced expression of iNOS mRNA correlates with reduced NO serum levels [12]. In the present study, the decrease of NO plasma level mediated by rosiglitazone (about 30%) contrasted the marked downregulation of iNOS (about 60%). One possible explanation may be that NO was measured indirectly by its metabolites, which are in part excreted by the kidneys. As deteriorated renal function is only in part restored by rosiglitazone, NO metabolites may accumulate even if iNOS expression is already markedly reduced. 

In addition to altering the transcriptional status, PPAR*γ* agonists can stimulate endothelial NO release by modulating posttranslational mechanisms of eNOS regulation. In vitro, rosiglitazone increases heat shock protein 90-eNOS interaction [35] and induces a phosphorylation of eNOS at Ser-1177 [36]. Both mechanisms result in a significantly increased production of NO. In our in vivo study it is possible that posttranslational modifications augmented the eNOS activity as well and contributed to an increased NO release.

Besides the influence on the transcriptional pathway by activating peroxisome proliferator-activated receptor, rosiglitazone is known to exert protective effects independent from PPAR. In ischemic stroke rosiglitazone is antiinflammatory by directly reducing Nf*κ*B activity and has antiapoptotic properties by preserving intracellular ATP levels [37]. Although high doses of rosiglitazone are necessary to elicit PPAR-independent responses [38] we cannot exclude that rosiglitazone has antiapoptotic and antiinflammatory effects in renal ischemia/reperfusion injury which are independent from PPAR*γ*. Consequently, these effects of rosiglitazone may additionally contribute to renal functional and morphological amelioration in I/R injury. An object for further studies will be the question whether the additional use of a specific PPAR*γ* antagonist like GW9662 can block the effects of rosiglitazone in renal I/R injury.

At the moment from the data given we have no explanation in detail why protection might be more pronounced after severe ischemia with extended clamping time. We can only speculate about this fact. One possible explanation could be that the extent of eNOS/iNOS imbalance seems to depend on duration of ischemic period. Chen et al. demonstrated that eNOS mRNA is not down, but upregulated after 45 min of ischemia and 24 h of reperfusion [8], whereas we showed a significant eNOS downregulation with 60 min ischemic period.

Beside eNOS/iNOS imbalance, other mediators are described to contribute to renal damage and dysfunction in I/R injury, for example, accumulation of free Ca^2+^ in cytosol and calpain, releasing of TNF-alpha and other cytokines or generation of oxygen radicals and generation of tubular casts [1].

These other multiple pathways involved in renal I/R injury and not affected by rosiglitazone may explain the contrast between the distinct improvement of altered inflammatory and apoptotic markers corresponding with the reduction of renal histomorphological damage and the rather moderate improvement of renal function by rosiglitazone.

One should take into consideration that a reperfusion period of 24 hours after a severe renal injury with 60 min of ischemia is a short time for restoring the plenitude of the complex renal functions. Previous studies of our group show that complete recovery of renal function after I/R injury with a clamping time of 45 minutes takes several days [4]. Based on that we speculate that an even more pronounced functional recovery due to rosiglitazone might take place when our findings of apoptotic and inflammatory infiltrative markers as well as histologic parameters are extrapolated on a prolonged subsequent follow-up time. This explanation could bridge the gap between the strong antiinflammatory, antiapoptotic properties, and the histopathologic improvement on the one hand and the moderate increase of renal function on the other hand. The effect of rosiglitazone on long-term functional renal recovery after severe I/R injury is therefore an interesting task for future studies.

## 5. Conclusion

We are the first to show that (i) rosiglitazone (5 mg/kg) applied in a single dose has functional and histomorphological beneficial effects in a model of severe ischemia (60 min)/reperfusion injury after a 24 h period of reperfusion. (ii) This improvement of renal outcome is linked to a reduction of increased inflammatory and apoptotic markers as well as a reversed eNOS mRNA downregulation and iNOS mRNA upregulation. We hypothesize that rosiglitazone improves renal outcome in I/R injury by rebalancing these key enzymes of the nitric oxide pathway (Figure 7).

## Abbreviations

(i)AKI: (Ischemic) acute kidney injury
GFR: Glomerular filtration rate
I/R: Ischemia/reperfusion
iNOS: Inducible nitric oxide synthase
eNOS: Endothelial nitric oxide synthase
PAH: Para-aminohippurate
RPF: Renal plasma flow
NO: Nitric oxide
PPAR*γ*: Peroxisome proliferator-activated receptor gamma
RGZ: Rosiglitazone
CC3: Cleaved caspase 3
PAH-NS: Para-aminohippurate net secretion
PTC: Renal proximal tubular cell.
